# Modified Conventional Teaching: An Assessment of Clinical Biochemistry Learning Process Among Medical Undergraduate Students Using the Traditional Teaching in Combination with Group Discussion

**DOI:** 10.7759/cureus.5396

**Published:** 2019-08-16

**Authors:** Sabitha Vadakedath, Venkataramana Kandi

**Affiliations:** 1 Biochemistry, Prathima Institute of Medical Sciences, Karimnagar, IND; 2 Microbiology, Prathima Institute of Medical Sciences, Karimnagar, IND

**Keywords:** clinical biochemistry, didactic lectures, conventional teaching method, teaching biochemistry, medical undergraduates, group discussion, multiple choice questions (mcq’s)

## Abstract

Background: Clinical biochemistry is a branch of organic chemistry which involves a detailed study on the normal functioning of living cells in relation to the disease. The subject is not only volatile but also complicated. Also, teaching biochemistry to medical undergraduates is really a challenging job. Routine didactic lectures alone may not be enough for students while learning clinical biochemistry.

Methods: This study included 90 first-year medical undergraduate students. They were grouped as three groups of 30 students each. The routine conventional classroom teaching method was followed by a group discussion session. The topics were informed in advance so that they were provided with adequate time to prepare and be mentally ready for the session. The group discussion was preceded by a set of 10 multiple-choice questions (MCQs), and a final assessment of five MCQs following the discussion.

Results: There was only minor difference in the marks obtained by various student groups after the group discussion with Group B students (5.5 ± 1.54; p < 0.001) performing better than the other two groups. Students also scored evenly in the final assessment using MCQs with Group A (2.7 ± 1.36; p < 0.001) performing better than the other two groups. Prior to the group discussion session, 33% of the students in all the groups scored more than 75% of marks. The overall performance of all the students after the final assessment using MCQs revealed that 60% of the students scored more than 75% marks.

Conclusion: The study results confirm the fact that the modified conventional teaching method appears to be better than traditional teaching. The student performances had significantly improved with such kind of education process. The results also highlight the importance of increased student efforts, probably including group discussions and revisions to improve understanding and subject retention.

## Introduction

Biochemistry is one of the three subjects included in the first year of medical undergraduate course along with the human anatomy and human physiology. It deals with understanding the basic functions of the body and its relation to the disease. The knowledge of which is essential for the better management of patients. Students pursuing a medical undergraduate course, although have a good knowledge of chemistry, they hardly have any idea of how the clinical biochemistry is as a subject. Teaching biochemistry to them in relation to the human body is a challenge to the medical teachers. Currently, there are various teaching methods that include the conventional lectures using the chalk and board, PowerPoint presentations, tutorials, demonstrations, seminars, group discussions, problem-based learning approach, etc. [1-2]. 

Teaching through lectures is monotonous and is a widely used teaching method for a large group of students. Tutorials, demonstrations, group discussions, and problem-based learning approach are generally used to teach small groups [3]. Other student-centered learning approaches appear to have been positively received by the students [4]. In a lecture for a large group of students, the teacher may not be able to understand individual student’s perspective regarding their subject retention. But during small group teaching including the discussions, demonstrations, tutorials, etc., the teacher can evaluate each student individually. Recent research suggests that student performances may be enhanced by including active learning sessions that include the group discussion [5].

Various learning approaches have recently been tried, which includes the idea of a flipped classroom where the students were initially asked to watch videos, and later during the traditional class, they were made to discuss the topic. Flipped classroom exposes the students to various learning and assessment processes that include the watching of lecture videos, online quizzes, discussion sessions, and the traditional evaluation [6-7]. 

A recent study had assessed the student perception towards the flipped classroom and had noted that most students showed satisfaction with the flipped classroom teaching over the traditional lecture-based teaching [8].

The present study is carried out to know the effectiveness of a large group conventional lecture by a teacher followed by a small group discussion and a final evaluation by multiple-choice questions (MCQs) to teach clinical biochemistry to first-year medical undergraduates.

## Materials and methods

A total of 90 students were enrolled in the study. The students were detailed about the study methodology and only those who showed interest were included in the study after oral consent. The study was also approved by the institutional ethical committee. All the 90 students were divided into three groups with 30 students each and were labeled as Groups A, B, and C.

Characteristics of student groups and the role of the teacher

The student grouping was random, which included a mix of varied academic performers. Each group was moderated by a teacher of Assistant Professor cadre and above. The student groups were balanced taking the age, sex/gender, and personalities into consideration. Each group had students with high, moderate, and low cognitive skills/capabilities. All the groups were adequately oriented regarding the functioning of the group discussion session. All the study participants were sensitized about active participation and not to compete during the process.

The teacher’s role was to moderate the students of various capabilities and to maintain harmony within the study groups. The teacher would provide the exact learning objectives, and also provide necessary resources that include the reference books, reliable internet resources, and other infrastructural facilities like the overhead projector.

The topics included for this study were carefully selected taking students/participants into consideration. The study included topics like the chemistry of carbohydrates, glycolysis, Kreb’s cycle (tri-carboxylic acid cycle), gluconeogenesis, HMP (hexose monophosphate) shunt pathway, vitamins, and detoxification.

**Figure 1:**
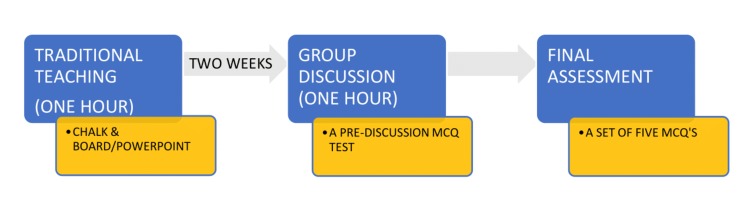
Diagrammatic representation of the study methodology. MCQS, multiple choice questions.

A traditional lecture was a part of the teaching curriculum, where the students were taught using chalk and board and/or PowerPoint. After two weeks of the lecture, a session of group discussion for one hour was planned. The topics for the discussion were intimated one week prior to the students, giving them adequate time for preparation. The details of the study methodology are depicted in Figure 1.

 

The department of biochemistry under the guidance of the head of the department, and with the help of the teachers participating in the study had prepared a set of 10 MCQs (pre-test MCQs) each carrying one mark for the evaluation of students prior to the group discussion session. Each question included in the questionnaire was provided with an answer key, which formed the basis of assessment by the teachers to rule out any discrepancy in evaluation.

During the discussion sessions, the essential learning points of the topics were elaborated by the moderator to the respective group. The students were made to discuss the topic among themselves and using the relevant textbooks and other online resources of biochemistry.

Following the discussion session, each student was asked to answer a set of five MCQs (final assessment) each carrying one mark. This questionnaire was used to assess the student’s cognitive skills at the end of each session.

The study results were tabulated in a Microsoft excel sheet and were analyzed by drawing percentages, mean, standard deviation (SD), and p values.

## Results

The student’s evaluation of pre-group discussion evaluation revealed an almost similar performance by all the groups as shown in Table 1.

**Table 1:** Pre-discussion assessment of the study groups. *Statistically significant; SD, standard deviation; p-value, calculated probability value.

Group	Mean ± SD	p-Value
A	4.86 ± 1.35	<0.001*
B	5.50 ± 1.54	<0.001*
C	5.39 ± 1.29	<0.001*

There was a slightly better performance by the Group B as compared to the Groups A and C. The final evaluation of the study groups using the MCQs revealed similar performances by all the groups as shown in Table 2.

**Table 2:** Final assessment results of the study groups. *Statistically significant; SD, standard deviation; p-value, calculated probability value.

Group	Mean ± SD	p-Value
A	2.70 ± 1.36	<0.001*
B	2.00 ± 1.17	<0.001*
C	1.89 ± 1.10	<0.001*

The performance evaluation of student groups after the pre-group discussion evaluation at different cut-offs (<50%; >50%; >75%) is shown in Table 3.

**Table 3:** Pre-discussion assessment of the study groups at different cut-offs.

Percentage of marks	A	B	C
<50%	7 (23.3%)	5 (16.7%)	2 (6.7%)
>50%	20 (66.7%)	21 (70%)	25 (83.3%)
>75%	3 (10%)	4 (13.3%)	3 (10%)

Final evaluation of the student groups based on the MCQs at different cut-offs (<50%; >50%; >75%) is shown in Table 4.

**Table 4:** Final assessment results of the study groups at different cut-offs.

Percentage of marks	A	B	C
<50%	14 (46.7%)	23 (76.7%)	25 (83.3%)
>50%	08 (26.7%)	01 (3.4%)	01 (3.4%)
>75%	08 (26.7%)	06 (20%)	04 (13.3%)

There was only a minor difference in the marks obtained by various student groups after the group discussion with Group B students (5.5 ± 1.54; p < 0.001) performing better than the other two groups. Students also scored evenly in the final assessment using MCQs with Group A (2.7 ± 1.36; p < 0.001) performing better than the other two groups.

At the pre-group discussion session, 33% of the students of all the groups scored more than 75% of marks. The overall performance of all the students after the final assessment using MCQs revealed that 60% of the students scored more than 75% marks.

## Discussion

Biochemistry is a volatile subject that includes complex reactions, which are not easy to remember. A teacher’s guidance assumes greater significance for the students to understand the subject. As it is a basic science subject having wide applications in all clinical specialties including the diagnostics, making the teaching/learning process effective appears to be a challenge for the teachers. The perception of students towards learning is also changing with the availability and access to information technology. In the present study, the students were exposed to the conventional lectures which were followed by a session of group discussion. They were finally evaluated using MCQs. The performances of the student groups in group discussion had improved the students’ overall subject retention as evidenced by the results of the final assessment, where 60% of all the students crossed more than 75% marks.

Increasing demand for a more effective teaching/learning process, especially of the basic sciences subjects including the biochemistry has brought in newer approaches to teach such subjects.

The inverted classroom teaching was recently suggested to teach biochemistry at the pre-clinical level of the medical undergraduate course. Here the students were given e-learning material prior to a classroom discussion, where the students were encouraged to discuss the topic with additional material and under the tutor’s moderation [9].

Due to the complexity of the basic science subjects, the students lack the motivation of learning the subject in the absence of knowledge of its practical applications, especially in-patient care perspective. Competency-based education could provide students with an understanding of the basic concepts of the subjects and its application to medicine [10].

The University of South Florida College of Medicine had previously implemented a program for fresh medical undergraduate students. This program was intended to stress the importance of skill development to successfully complete medicine studies. It also underlines the clinical significance of basic science subjects, continuous learning, ethics, and professionalism in medicine [11].

Only classroom teaching may not completely satisfy the students learning objectives. Recently a study had emphasized the importance of active engagement in the subject that includes seminars and discussions to improve the learning outcomes, especially with the basic science subjects [12].

A university study from Serbia assessed the role of computer simulation experiments, continual evaluation, oral tests along with the routine lectures to understand the basic animal physiology [13]. Complementing the current curriculum with advanced/innovative teaching aids like using the “Foldit puzzles” was suggested to teach biochemistry and biology up to the undergraduate level [14].

Engaging the students in interactive sessions like the group discussions was noted to improve the conceptual understanding of subjects like chemistry [15]. As biochemistry also involves several biochemical reactions and complicated pathways, conducting regular group discussion discourses would positively influence students learning objectives.

Previous research had also highlighted the significance of motivating students by using various instructional strategies that include open-atmosphere classroom teaching, conducting interactive sessions, guiding, and reminding students [16].

Computational techniques were used to visualize the three-dimensional structures of proteins and deoxyribose nucleic acid (DNA), along with tutorials to make biochemistry learning easy [17].

A study at the University of São Paulo had explored the idea of using published research articles to teach metabolism instead of didactic lectures and seminars. This study used a combination of directed study along with the group discussion [18].

Effectiveness of team-based learning (TBL) to teach the principles of medical biochemistry was positively evaluated by a study from Korea [19].

## Conclusions

Traditional didactic lectures may not completely be sufficient in the students learning with respect to clinical biochemistry. Group discussions among the students not only help in exchanging their opinions on the topic but also could provide a platform for a better understanding ability. As the students were sensitized about the topic of discussion, it created an opportunity for a healthy discussion. Also, the discussion was moderated effectively by the designated faculty, thereby enhancing the overall performances by the students. The students expressed the ease in understanding the subject biochemistry when they were exposed to interactive teaching methods. Thus, it can be concluded that biochemistry and probably other basic science subjects must be taught using different teaching methods i.e., traditional lectures combined with group discussions, tutorials, problem-based approach, and seminars. Biochemistry taught using combined teaching methods appear to positively impact students and enhance their learning ability and subject retention.
